# Bradycardia, Renal Failure, Atrioventricular-nodal Blocker, Shock, and Hyperkalemia Syndrome Diagnosis and Literature Review

**DOI:** 10.7759/cureus.6985

**Published:** 2020-02-13

**Authors:** Yasar Sattar, Syeda Beenish Bareeqa, Hiba Rauf, Waqas Ullah, M. Chadi Alraies

**Affiliations:** 1 Internal Medicine, Icahn School of Medicine at Mount Sinai, New York, USA; 2 Internal Medicine, Jinnah Medical and Dental College, Karachi, PAK; 3 Internal Medicine, Dow Medical College, Karachi, PAK; 4 Internal Medicine, Abington Hospital-Jefferson Health, Abington, USA; 5 Cardiology, Detroit Medical Center, Detroit, USA

**Keywords:** brash syndrome, bradycardia, av nodal blockers, shock, hyperkalemia

## Abstract

The combination of bradycardia, renal failure, atrioventricular (AV)-nodal blocker medications, shock, and hyperkalemia (BRASH) is a new syndrome that is a consequence of a positive loop of bradycardia due to AV-nodal blockers and hyperkalemia secondary to renal insufficiency. We present a case of BRASH syndrome in which the patient on chronic AV-nodal blockers presented with bradycardia, hypotension, underlying kidney dysfunction, and hyperkalemia. The patient was medically managed and discharged upon clinical improvement. The purpose of this report is to highlight the rare cases of BRASH syndrome and improve its management.

## Introduction

Bradycardia, renal failure, atrioventricular (AV)-nodal blocker medications, shock, and hyperkalemia (BRASH) is a recently described syndrome by Josh Farkas in 2016. The syndrome results in severe bradycardia and hypotension due to the synergistic overlap of both AV-nodal blocker and hyperkalemia secondary to underlying renal failure. It can be triggered by risk factors that increase potassium, including fever, sepsis, medications, tumor lysis, renal insufficiency, diabetes, and hypovolemia. The investigations are centered on the complete metabolic panel and electrocardiogram. In cases where the patient presenting with profound bradycardia and shock does not respond to initial treatment, a diagnosis of BRASH syndrome is likely [1].

## Case presentation

A 66-year-old Caucasian woman was brought in by emergency medical services for concerns of severe lightheadedness that had progressed to a near syncope episode the night before. Prior to her arrival, she had expressed concerns of constant mild lightheadedness for one day, which progressed in severity and prompted her transfer to the hospital. Upon her arrival, she continued to have symptoms but denied any loss of consciousness, seizure, vision blackout, sweating, nausea, vomiting, or diarrhea.

Upon her admission, she was observed for vital derangements. Her vital signs showed marked bradycardia with a heart rate of 35 beats/minute, a blood pressure of 87/62 mmHg on standing and 90/65 lying down, a temperature of 98.3°F (38.8°C), a respiratory rate of 24 breaths/minute, and an oxygen saturation of 99% on 4 L/min of nasal cannula oxygen. Physical examination showed a well-nourished and afebrile patient in non-acute distress with dry skin and decreased skin turgor. She had a regular sinus rhythm with normal heart sounds on cardiovascular exam. Both lungs were clear on auscultation with normal breath sounds. Her abdomen was soft and non-tender with no positive findings.

Her past medical history was significant for hypertension, hyperlipidemia, diabetes mellitus type 2, coronary artery disease status with post-coronary intervention performed in 2015. She was taking aspirin 81 mg once daily (OD), atorvastatin 40 mg OD, carvedilol 12.5 mg twice daily (BID), clopidogrel 75 mg OD, losartan 25 mg OD, and metformin-sitagliptin 500-50 mg BID.

**Figure 1:**
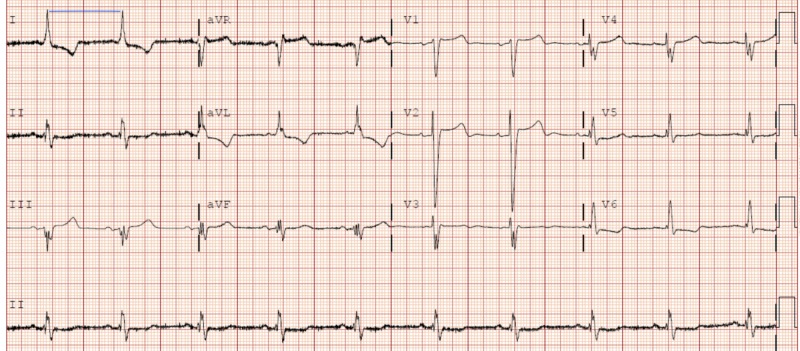
Electrocardiogram showing sinus bradycardia with an expected rate of 55 bpm

Blood work was negative except for high brain natriuretic peptide at 1,620 pg/mL, creatinine 2.21 mg/dL, blood urea nitrogen 34 mg/dL, troponin 0.811 and 1.66 ng/mL, potassium 6.2 mEq/L, and lactate 5.3 mmol/L. The electrocardiogram (EKG) findings are shown in Figure 1.

A constellation of symptomatic bradycardia with EKG findings, a baseline renal dysfunction, hyperkalemia, and an intake of the usual long-term dose of carvedilol suggested a preliminary diagnosis of BRASH. This syndrome precipitated due to a combination of carvedilol and hyperkalemia secondary to acute kidney injury. Treatment was started with an intravenous bolus of 1 L normal saline, calcium gluconate dose of 0.4 mEq/kg, and insulin 10 units with 50 mL/D50. The patient’s heart rate improved to normal with symptomatic resolution after one round of treatment.

## Discussion

This case highlights the importance of BRASH syndrome as a new diagnosis to consider. It is important to understand the physiology of BRASH as the advanced cardiovascular life support (ACLS) bradycardia algorithm generally does not work for BRASH treatment [1]. Hyperkalemia is a “hema-panic call” for physicians but can provide helpful information in some patients with bradycardia, as they have a positive loop of hyperkalemia causing worsening of AV-nodal depression if there is concomitant negative chronotropic drug intake.

Despite numerous cases with similar clinical presentations reported in medical literature, we have observed a paucity in recognition of it as a distinct syndrome. We performed extensive searches in the published medical literature from 2009 through 2019 to identify this anonymous clinical presentation. It was noticed that many clinicians have not clearly mentioned this clinical presentation as BRASH syndrome. Nine cases were identified in seven different studies [2-8]. The age of presentation ranged between 24 and 97 years with the mean age of 72 years. In our case, the presenting patient was 66 years old, which is around the mean age of previously presenting patients. Of the nine patients, female patients were predominant (78%) while only two were male patients (22%). The heart rate in patients with bradycardia varies from severe bradycardia (28 bpm) to relatively mild bradycardia (56 bpm) with a median of 33 bpm. However, the heart rate was 34 bpm in our patient.

All the patients in discussed cases of BRASH syndrome showed signs of renal failure. AV blockade was observed in seven of nine cases. An experimental study on canines reported that significant EKG changes, suggestive of AV-nodal blockade, are associated with hyperkalemia of ≥6.3 mEq/L [9]. We have observed a similar correlation in previously reported studies in human patients. Five cases reported serum potassium levels of ≥6.3 mEq/L (55.5%), while four patients had lower potassium levels. All patients with serum potassium levels of ≥6.3 mEq/L had concurrent AV blockade, while half of the patients with serum potassium levels of <6.3 mEq/L showed no sign of AV blockade on EKG. Cardiac electrical conduction abnormalities are also associated with other metabolic instabilities such as acidosis [10]. Our patient had significant hyperkalemia (6.2 mEq/L) with concurrent metabolic acidosis, which explains the EKG changes observed in this case. The details of previously reported anonymous cases on BRASH syndrome are given in Table 1.

**Table 1:** Chart review of studies reporting bradycardia, renal failure, atrioventricular-nodal blocker, shock, and hyperkalemia syndrome from the past decade

Study	Patient’s Age (Years)/Sex	Bradycardia	Renal Failure	Atrioventricular Blockade	Shock	Hyperkalemia	Correction
Simmons and Blazar 2019 [2]	24 years/male	40 beats/minute	Yes	Yes	No	7.4 mEq/L	Atropine sulfate, calcium gluconate, pacemaker
Ahmad and Tan 2017 [3]	81 years/female	33 beats/minute	Yes	Yes	No	5.8 mEq/L	Intravenous atropine, isoproterenol
Juvet et al. 2013 [4]	85 years/female	33 beats/minute	Yes	Yes	Yes	10.1 mEq/L	Insulin, calcium gluconate, bicarbonates, hemodialysis
Hegazi et al. 2012 [5]	65 years/female	48 beats/minute	Yes	No	No	5.6 mEq/L	Calcium gluconate, insulin
	57 years/female	44 beats/minute	Yes	No	No	5.5 mEq/L	Calcium gluconate
Aziz et al. 2011 [6]	97 years/female	56 beats/minute	Yes	Yes	No	6.3 mEq/L	Calcium chloride, normal saline, insulin
	86 years/female	30 beats/minute	Yes	Yes	Yes	5.7 mEq/L	Atropine, normal saline, pacemaker, insulin
Erden et al. 2010 [7]	76 years/female	28 beats/minute	Yes	Yes	No	9.2 mEq/L	Temporary percutaneous pacing, bicarbonate, insulin
Argulian 2009 [8]	79 years/male	28 beats/minute	Yes	Yes	No	6.4 mEq/L	Calcium gluconate, bicarbonate, insulin, sodium polystyrene sulfonate

The medication history of AV-nodal blockers with the clinical evidence of hyperkalemia is essential to make the diagnosis of BRASH syndrome. In previous studies, the most used AV-nodal blockers that resulted in BRASH syndrome were β-blockers and calcium-channel blockers. These data are tabulated in Table 2.

**Table 2:** Atrioventricular-nodal blockers that precipitated bradycardia, renal failure, atrioventricular nodal blocker, shock, and hyperkalemia syndrome in each case

Study	Atrioventricular-Nodal Blocker	Drug Class
Simmons and Blazar 2019 [2]	Metoprolol	β-Blocker
Ahmad and Tan 2017 [3]	Amlodipine, bisoprolol	Calcium-channel blocker, β-blocker
Juvet et al. 2013 [4]	Sotalol	Combine K^+^-channel and β-blocker
Hegazi et al. 2012 [5]	Verapamil	Calcium-channel blocker
	Verapamil	Calcium-channel blocker
Aziz et al. 2011 [6]	Amlodipine	Calcium-channel blocker
	Carvedilol	Non-selective β-blockers
Erden et al. 2010 [7]	Carvedilol	Non-selective β-blockers
Argulian 2009 [8]	Amlodipine, metoprolol	Calcium-channel blocker, β-blocker

In order to make a diagnosis of BRASH syndrome, the patient should have bradycardia in the setting of synergistic effect of hyperkalemia and history of use of AV-nodal blockers with underlying renal insufficiency secondary to any medical condition. Mostly, BRASH patients are on the usual chronic doses of chronotropic drugs and often do not have signs of a drug overdose. They are seen to be hyperkalemic and have a certain degree of renal insufficiency. AV-nodal blockers concomitant with hyperkalemia can cause any type of bradycardia ranging from junctional bradycardia to third-degree AV block. Patients with BRASH syndrome may present with severe symptomatic bradycardia with a mild elevation of potassium without any EKG findings of hyperkalemia. Most of the patients clinically respond to intravenous calcium 40 mg/kg and insulin with D50.

## Conclusions

BRASH syndrome presents with severe hypotension and bradycardia in a setting of chronotropic drug use and renal failure. Patients should be evaluated comprehensively for possible risk factors for hyperkalemia. The initial treatment should be directed toward improving the potassium levels and correcting the volume status. In refractory cases, catecholamines and a pacemaker may be needed to improve the hemodynamic status; however, the ACLS algorithm is not effective in managing these cases. Clinicians should take a careful approach when prescribing AV-nodal blockers in patients with multiple comorbidities and underlying kidney dysfunction.
